# Identification of Semiochemicals from Cowpea, *Vigna unguiculata*, for Low-input Management of the Legume Pod Borer, *Maruca vitrata*

**DOI:** 10.1007/s10886-020-01149-7

**Published:** 2020-01-17

**Authors:** Jonathan Osei-Owusu, József Vuts, John C. Caulfield, Christine M. Woodcock, David M. Withall, Antony M. Hooper, Samuel Osafo-Acquaah, Michael A. Birkett

**Affiliations:** 1 Department of Chemistry, Kwame Nkrumah University of Science and Technology, Kumasi, Ghana; 2 Biointeractions and Crop Protection Department, Rothamsted Research, Harpenden, AL5 2JQ UK; 3 School of Biological and Chemical Sciences, Queen Mary University of London, London, UK

**Keywords:** Cowpea, *Vigna unguiculata*, Legume pod borer, *Maruca vitrata*, Semiochemicals, Natural enemies, IPM

## Abstract

Cowpea, *Vigna unguiculata* L. Walp. (Fabaceae), is one of the most important food legumes grown on the African continent, as it provides an affordable source of dietary protein. Yields of cowpea are significantly reduced through damage by legume pod-borer, *Maruca vitrata* (Lepidoptera: Crambidae)*,* caterpillars to flowers, tender leaves and pods. Semiochemical-based strategies are considered as environmentally benign and affordable for pest management, particularly on smallholder farms. In this study, we investigated the importance of cowpea flower volatiles as host location cues for egg-laying *M. vitrata*, and herbivore-induced plant volatiles (HIPVs) as *M. vitrata* repellents and natural enemy (*Apanteles taragamae* and *Phanerotoma syleptae* parasitoid) attractants. In oviposition choice assays, *M. vitrata* laid more eggs on flowering cowpea plants than non-flowering plants. Coupled gas chromatography-electrophysiology (GC-EAG) analysis using the antennae of female *M. vitrata* and an extract of flower volatiles collected by dynamic headspace collection revealed the presence of five EAG-active components that were identified by coupled GC-mass spectrometry (GC-MS) analysis as benzaldehyde, benzyl alcohol, acetophenone, a vinylbenzaldehyde isomer and (*E*)-cinnamaldehyde. A synthetic blend of the identified compounds, prepared using 3-vinylbenzaldehyde, induced *M. vitrata* to lay as many eggs on non-flowering cowpea as on flowering plants. The moths also preferred laying eggs on intact plants compared to *M. vitrata*-infested plants. As the emission of EAG-active floral compounds was determined to be lower in the headspace of infested cowpea flowers, the role of HIPVs emitted by *M. vitrata*-damaged leaves was also investigated. Of the compounds induced by larval damage, (*E*)-DMNT, indole, n-hexyl acetate, 1-octen-3-ol and linalool were shown by GC-EAG to possess electrophysiological activity. A synthetic blend of the EAG-active compounds, using racemic 1-octen-3-ol and linalool, significantly reduced egg numbers on flowering cowpea. Larval and egg parasitoids, i.e. *A. taragamae* and *Ph. syleptae,* respectively, of *M. vitrata* both preferred the Y-tube olfactometer arm treated with synthetic (*E*)-DMNT, whereas preference for racemic linalool and (*E*)-nerolidol was dose-dependent in *A. taragamae*. Our results provide the platform for the development of future semiochemical-based pest management strategies against *M. vitrata* on smallholder farms in West Africa.

**Electronic supplementary material:**

The online version of this article (10.1007/s10886-020-01149-7) contains supplementary material, which is available to authorized users.

## Introduction

Cowpea, *Vigna unguiculata* L. Walp. (Fabaceae), is one of the most important food legumes grown on the African continent, as it provides an affordable source of dietary protein (Addo-Quaye et al. 2011; Agyeman et al. 2014). The grain contains about 25% protein and 64% carbohydrate (FAO, 2004), and provides a source of minerals, vitamins and essential micronutrients (Boukar et al. 2013; Togola et al. 2017). However, crop yields remain very low, which is mainly due to biotic and abiotic factors including pest insects, diseases, parasitic weeds, drought and low soil fertility (Singh and Jackai 1985). The legume pod-borer, *Maruca vitrata* Fabricius (syn. *M. testulalis* Geyer) (Lepidoptera: Pyralidae), negatively affects the production of cowpea and other legumes not just in sub-Saharan Africa, but also in Asia and Latin America (Sharma et al. 1999). Larval feeding damage to cowpea plants occurs on flower buds, flowers, seed pods, tender leaves and terminal shoots, and losses in grain yield of 20–80% have been estimated from infestation by this pest alone (Sharma et al. 1999; Singh et al. 1990; Machuka et al. 1999). The cryptic feeding habit of the larvae protects them from contact insecticides, which then often results in insecticide overuse and the subsequent development of insecticide resistance (Downham et al. 2004; Ekesi 1999). Alternative leguminous host plants of *M. vitrata* include *Sesbania cannabina* (Retz.) Pers., which can adapt well to drought, water logging, high alkalinity, metal toxicity, and is used as a green manure crop to help fix atmospheric nitrogen (Funakoshi et al. 2015; Huang et al. 2003; Nitisha 2014).

In search of non-insecticidal-based strategies for *M. vitrata* management, breeding resistant cowpea varieties with traits affecting pod wall thickness, trichomes, nutritional and antibiotic content has been attempted, but to date, no cowpea line has been identified to possess the desired levels of resistance to the pest (Fatokun et al. 2002). A transgenic cowpea variety expressing the Bt-protein Cry1Ab against *M. vitrata* already exists, but is unavailable for use by African farmers (Oyewale and Bamaiyi 2013). More sustainable, low-input management strategies include the deployment of semiochemicals that *M. vitrata* uses to find suitable hosts, mates, oviposition substrates, or to escape competition and natural enemies, and avoid toxic plant defense chemicals. The female sex pheromone components of *M. vitrata* have been identified as *(E,E)-*10,12-hexadecadienal, *(E,E)-*10,12-hexadecadienol and *(E)-*10-hexadecenal, and are used for monitoring populations (Adati and Tatsuki 1999; Downham et al. 2004; Schläger et al. 2015). Although the synthetic pheromone blend can attract 11–50% females of the total catch (Downham et al. 2004), the female ratio could be further increased to provide more precise forecasts for the timing of egg-laying (Wall 1989). Because volatile cues from the larval host can be key for ovipositing insects to locate suitable plants (Städler 1994), cowpea volatile blends serve as potent kairomones for host-seeking *M. vitrata*. Feng et al. (2017) found (*E*)-(1*R*,9*S*)-caryophyllene to induce egg-laying on cowpea, and Wang et al. (2014) described the blend of limonene, 1,3-diethylbenzene, 4-ethylbenzaldehyde and 4-ethylacetophenone as an attractant for females.

When used concurrently, attractive and repellent volatile blends can enhance the effectiveness of semiochemical-based management strategies (e.g. Hassemer et al. 2019). Volatiles from conspecific-infested host plants can be the source of such repellent compounds, as demonstrated in the case of *Heliothis virescens* Fabr. (Lepidoptera: Noctuidae) (De Moraes et al. 2001), for example, where egg-laying females were repelled by the blend of their host plant, *Nicotiana tabacum* L. (Solanaceae), released in response to feeding by conspecific caterpillars. Furthermore, natural enemies of the pest can also utilize herbivore-induced plant volatiles (HIPVs) as kairomones to locate prey (e.g. Aartsma et al. 2017). The prospect of biological control with the solitary larval endoparasitoid *Apanteles taragamae* Viereck (Hymenoptera: Braconidae), which is capable of parasitising on average 63% of *M. vitrata* larvae on *S. cannabina* (Huang et al. 2003), led to studies on exploiting this natural enemy for biological control of *M. vitrata* feeding on cowpea (Dannon et al. 2010). Also, another braconid, the egg parasitoid *Phanerotoma syleptae* Zettel, was recently introduced into Benin from Asia for the control of *M. vitrata* (Srinivasan et al. 2015). In view of the potential for using host-derived semiochemicals for insect pest management, the aims of the current work were four-fold: 1) to identify volatile compounds from cowpea that attract *M. vitrata* for egg-laying; 2) to identify HIPVs from cowpea that repel egg-laying *M. vitrata*; 3) to identify HIPVs produced by *S. cannabina* in response to *M. vitrata* larval feeding and compare them with HIPVs from cowpea; and 4) to assess olfactory responses of the parasitic wasps *A. taragamae* and *Ph. syleptae* to selected cowpea HIPVs*.* Characterization of semiochemicals that deter the pod borer or induce a change in cowpea defence status to attract parasitoids would provide the basis for novel *M. vitrata* management strategies on smallholder farms using either synthetic or plant-derived attractants and repellents.

## Methods and Materials

### Plants

Seeds of the Ghanaian cowpea cultivar Padi-tuya were obtained from the Savanna Agriculture Research, Tamale, Ghana. One seed per pot (9 × 9 × 10 cm) was grown in a greenhouse at Rothamsted Research, UK (27 °C:25 °C and 16:8 h L:D photoperiod, LED lighting) in soil (pH 5.5–6.0; 75% medium-grade peat, 12% sterilised loam, 3% medium-grade vermiculite; 10% 5 mm lime-free grit; <2 mm particle size after sieving). Seeds of *S. cannabina* were provided by the International Institute of Tropical Agriculture (IITA), Benin, for experimental use. One seed per pot (20 × 30 cm) was grown in heat-sterilized loamy soil at 36 °C:23 °C and 12:12 h L:D photoperiod in a greenhouse at the Kwame Nkrumah University of Science and Technology (KNUST), Kumasi, Ghana.

### Insects

*Maruca vitrata* colonies were reared at KNUST at 26 °C, with a photoperiod of 12:12 L:D and 76% relative humidity. Five-day-old, mated adult females were transferred into transparent cylindrical plastic cups (3 cm diameter × 3.5 cm height) for oviposition for 48 h, and kept on 10% honey solution on a piece of filter paper. Cups with eggs were subsequently incubated in a large plastic container with sprouting cowpea grains as feeding substrate for newly hatched larvae, which were replaced with new grains every three days until pupation. Pupae were collected from the diet after 15 days and were placed inside a netted cage under similar conditions for emergence. Fifth instar larvae of *M. vitrata* were transported to Rothamsted Research, UK, where they were placed in open petri dishes with artificial diet, prepared according to Jackai and Raulston (1988) and provided by IITA, Nigeria [400 g cowpea flour, 127.2 g wheatgerm, 44.4 g Wesson salt mix, 25 g ascorbic acid, 3.9 g aureomycin, 60 g sugar, 3.6 g methyl *p*-hydroxybenzoate, 6.8 g sorbic acid, 2 L water, 22 mL potassium hydroxide (4 M aq. solution), 29.6 mL choline chloride (15%), 50 mL acetic acid (25%), 26 mL formaldehyde (10%), 30 mL vitamin suspension and 59.2 g agar] and kept in a netted cage at 25 °C:22 °C and 12:12 h L:D photoperiod. Emerged adults were fed with 10% sugar solution. After five days, mated females were isolated in groups of two in transparent plastic cups for two days for oviposition. Cups with eggs were placed on artificial larval diet. A colony of the larval endoparasitoid *Apanteles taragamae* was started by obtaining cocoons from a stock culture maintained at IITA, Benin. Subsequent colonies were produced by exposing first instar *M. vitrata* caterpillars (two days old) to parasitization by 3-day-old female *A. taragamae* for 24 h*.* Parasitized caterpillars were reared on sprouting cowpea grains until pupation. Cocoons of *A. taragamae* were collected from the sprouting diet after seven days of parasitism and were placed inside a netted cage for emergence. Emerge adults were fed with honey solution streaked inside the cage. Cocoons of the egg parasitoid *Phanerotoma syleptae* were also obtained from IITA, Benin. Emerged adults were fed with honey solution streaked inside the walls of the rearing cage. To allow mated wasps to parasitize hosts, *M. vitrata* eggs in small cylindrical cups (3 cm diameter × 3.5 cm height) were offered to 2-day-old mated *P. syleptae* females*.* Parasitized larvae were reared on sprouting cowpea diet as described above.

### Collection of Volatiles by Dynamic Headspace Sampling (Air Entrainment)

Plant material was enclosed in glass vessels and connected with a charcoal-cleaned air source (Capillary-Grade Hydrocarbon Trap with 1/8 in. compression fittings; Thames Restek Ltd., High Wycombe, UK), supplying an inflow of 700 mL/min, which was then drawn through a Porapak Q trap (50 mg polymer load, 50/80 mesh, Supelco, Bellefonte, PA) at 600 mL/min at the air outlet (T = 22 °C; Pye volatile collection kits, Kings Walden, UK). Prior to use, glass vessels and metal plates were washed with detergent (Teepol), acetone and distilled water, and baked overnight at 140 °C. Porapak Q tubes were conditioned before use by washing with 4 mL distilled diethyl ether and heating at 132 °C under a stream of nitrogen. Adsorbed volatiles were eluted from the polymer by washing with 750 μL of redistilled diethyl ether and then concentrated to 50 μL under a gentle stream of nitrogen gas. The experiments were replicated four times. Intact plants and empty glass chambers served as control.

### Collection of Cowpea Volatiles from Flowers

Two cowpea flowers were enclosed in a glass vessel (100 mm i.d. × 60 mm height) attached to semicircular aluminum plates around the plant stem, and air entrainment was run for 24 h. For the infestation experiment, two *M. vitrata* larvae (2nd instar) were carefully placed on two cowpea flowers at night. The larvae were then given 30 min to settle before entraining the flowers for 24 h.

### Collection of Volatiles from Leaves of Cowpea and *S. cannabina*

For the isolation of HIPVs, ten 2nd/3rd instar larvae were placed on the tender leaves of two-to-three-week-old cowpea plants with no flowers. Each of the plants was then enclosed in a glass vessel (95 mm i.d × 210 mm height), the bottom of which fitted around the plant stem by using two semicircular aluminum plates with a hole in the centre. To simulate the effect of mechanical damage during caterpillar feeding on cowpea volatile emission, five holes per leaf were made on three leaves of a cowpea plant, using a standard A4 paper hole punch, imitating the extent of damage caused by *M. vitrata* caterpillars. Air entrainments started immediately after the mechanical damage and ran for 6 h. For *S. cannabina*, leaves of three-week-old plants were infested with ten *M. vitrata* larvae (2nd instar), enclosed as above, and HIPVs collected for 72 h.

### Coupled Gas Chromatography (GC)-Electroantennography (GC-EAG)

Electrophysiological responses from the antennae of 5-day-old mated female *M. vitrata* moths to extracts of cowpea leaf volatiles were recorded using coupled GC-electrophysiology (Wadhams 1990). A moth was immobilized by chilling on ice, one of its antennae removed and the tip of the last segment cut off to ensure a good contact. The excised antenna was then mounted between two glass capillary electrodes filled with ringer solution (7.55 g/L sodium chloride, 0.64 g/L potassium chloride, 0.22 g/L calcium chloride, 1.73 g/L magnesium chloride, 0.86 g/L sodium bicarbonate, 0.61 g/L sodium orthophosphate). Antennal signals were passed through a UN-06 high-impedance amplifier (Ockenfels Syntech GmbH, Germany). Separation of the volatiles collected from cowpea was achieved on an Agilent 6890 N GC (Agilent Technologies), equipped with a cool on-column injector and a flame ionization detector (FID), using a 50 m × 0.32 mm i.d., 0.52 μm film thickness HP-1 column. The oven temperature was maintained at 30 °C for 2 min and then programmed at 5 °C/min to 250 °C. The carrier gas was helium. The outputs from the EAG amplifier and the FID were monitored simultaneously and analysed using a customised software package (Syntech GC/EAD for Windows v 2.3 09/1997). One μL aliquots of the pooled volatile samples were analyzed. A compound was identified as EAG-active if it evoked an antennal response in all of the three coupled runs for both the flower and leaf headspace extracts.

### GC Analysis and Coupled GC-Mass Spectrometry (GC-MS)

Volatile extracts were analyzed on a GC (Agilent Technologies, 6890 N, Stockport, UK), equipped with an FID and a HP-1 capillary column (50 m × 0.32 mm i.d., 0.52 μm film thickness). The oven temperature was maintained at 30 °C for 1 min and programmed at 5 °C/min to 150 °C, where it was held for 0.1 min, then at 10 °C /min to 230 °C and held for 27 min. The carrier gas was hydrogen. One μL of sample was injected into the injection port of the equipment manually. GC-MS analysis of eluted volatiles was performed using a Waters Autospec Ultima mass spectrometer couple to an Agilent 6890 GC fitted with a HP-1 capillary column (50 m × 0.32 mm id, 0.52 μm film thickness). Ionization was by electron impact (70 eV, source temperature 220 °C). Helium was the carrier gas. The oven temperature was maintained at 30 °C for 5 min, and then programmed at 5 °C/min to 250 °C. Tentative identifications were made by comparison of mass spectra with NIST 2005 mass spectral database. Confirmation of peak identity was made by comparison of their Kováts index (KI) values and GC peak enhancement with authentic compounds. Compounds were quantified using the single point external method with an n-alkane (C_7_-C_22_) mixture. Statistical analysis of cowpea volatile data (ng/h) was done using two-sample *t*-test on log-transformed values (GenStat version 16).

### Chemicals

Benzyl alcohol (99%) was obtained from Sigma-Aldrich, USA. (*E*)-Nerolidol (analytical standard), (*E*)-cinnamaldehyde (99%), acetophenone (99%), 3-vinylbenzaldehyde (97%), benzaldehyde (99%), (*Z*)-3-hexenyl acetate (>95%), (*E*)-2-hexenal (98%), (*Z*)-3-hexen-1-ol (98%), n-hexyl acetate (99%), (*RS*)-limonene (97%), methyl salicylate (99%), decanal (97%), myrcene (analytical standard) were purchased from Sigma-Aldrich, UK. (*RS*)-1-Octen-3-ol (98%) was obtained from Alfa Aesar, UK, (*RS*)-linalool (97%) was from Fluka, Switzerland, and indole (99%) was from Avocado Research Chemicals, UK. (*E*)-Ocimene was synthesized via the synthetic route previously reported from our group (Hassemer et al. 2016). Nonanal and decanal were purified by dissolving one gram of each compound in pentane (20 mL) and washing three times with saturated NaHCO_3_ solution. The organic layer was dried (MgSO_4_) and concentrated under vacuum to yield the purified compounds. (*E*)-4,8-Dimethyl-1,3,7-nonatriene[(*E*)-DMNT)] was synthesized as below. To a solution of geraniol (1.00 g, 6.48 mmol) in dichloromethane (20 mL) was added freshly prepared manganese dioxide (2.82 g, 32.40 mmol) and the mixture stirred for 24 h. The mixture was diluted with dichloromethane (20 mL), filtered through celite and concentrated under vacuum to give geranial (0.96 g, 97% yield) as a colourless oil. ^1^H-NMR (CDCl_3_, 500 MHz): δ 10.01 (d, 1H, *J* = 8.1 Hz, C**H**O), 5.90 (d, 1H, *J* = 8.1 Hz, CHOC**H**), 5.09 (t, 1H, *J* = 6.7 Hz, (Me)_2_C=C**H**), 2.27–2.21 (m, 4H), 2.19 (s, 3H, CHOCH=C(**Me)**), 1.71 (s, 3H, (**Me**)_2_C=CH), 1.63 (s, 3H, (**Me**)_2_C=CH); ^13^C-NMR (CDCl_3_, 125 MHz): δ 191.35, 163.90, 132.96, 127.43, 122.57, 40.62, 25.74, 25.67, 17.74, 17.60. To a solution of methyl triphenylphosphonium bromide (2.81 g, 7.85 mmol) in THF (30 mL), cooled to 0 °C under N_2_, was added *n*-butyl lithium (5.1 mL, 8.24 mmol) and the solution allowed to warm to RT over 60 min. The mixture was cooled to −78 °C before geranial (0.96 g, 6.28 mmol) in THF (10 mL) was added. After stirring for 60 min, the mixture was allowed to warm to RT over 16 h. Diethyl ether (40 mL) was added and the mixture cooled to −20 °C for a further 30 min. The suspension was filtered through celite, the precipitate washed with ice-cold diethyl ether and the combined organic filtrates concentrated under vacuum. The crude product was purified on silica gel (100% pet ether) to give (*E*)-DMNT (0.92 g, 97% yield) as a colourless oil. ^1^H-NMR (CDCl_3_, 500 MHz): δ 6.61 (m, 1H, H_2_C=C**H**), 5.89 (d, 1H, *J* = 11.0 Hz, H_2_C=CHC**H**), 5.13 (m, 2H), 5.01 (d, 1H, *J* = 10.4 Hz, **H**_2_C=CH), 2.14 (m, 2H), 2.10 (m, 2H), 1.80 (s, 3H, H_2_C=CHCHC(**Me**)), 1.71 (s, 3H, (**Me**)_2_C=CH), 1.64 (s, 3H, (**Me**)_2_C=CH); ^13^C-NMR (CDCl_3_, 125 MHz): δ 139.58, 133.44, 131.74, 125.40, 123.95, 114.57, 39.88, 26.52, 25.72, 17.70, 16.68. (*E*)-β-Farnesene was synthesized as below. To a solution of (*E,E*)-farnesol (1.00 g, 4.5 mmol) and 3,4-dihydropyran (1.89 g, 22.50 mmol) in dichloromethane (30 mL) was added *p*-toluenesulfonic acid (86 mg, 0.45 mmol), and the mixture was stirred for 60 min. The reaction mixture was diluted with dichloromethane before being washed with water, sat NaHCO_3_, dried (MgSO_4_) and concentrated under vacuum. The crude product was purified on silica gel (4% Et_2_O in pet ether) to give THP-farnesol (1.10 g, 80% yield) as a colourless oil. ^1^H-NMR (CDCl_3_, 500 MHz): δ 5.39 (m, 1H, OCH_2_C**H**=C), 5.12 (m, 2H), 4.65 (m, 1H, OC(CH_2_)**H**O), 4.26 (dd, 1H, OC**H**_2_CH=C, *J*_*1*_ *=* 11.9 and 6.4 Hz), 4.05 (dd, 1H, OC**H**_2_CH=C, *J =* 11.9 and 7.5 Hz), 3.92 (m, 1H, OC**H**_2_CH_2_), 3.53 (m, 1H, OC**H**_2_CH_2_), 2.15–2.00 (m, 8H), 1.84 (m, 1H), 1.74 (m, 1H), 1.71 (m, 6H), 1.62–1.54 (m, 9H); ^13^C-NMR (CDCl_3_, 125 MHz): δ 140.33, 135.27, 131.35, 124.35, 123.91, 120.57, 97.81, 63.67, 62.31, 39.66, 30.74, 26.74, 26.31, 25.73, 25.52, 19.65, 17.71, 16.45, 16.04. To a solution of THP-farnesol (1.10 g, 3.59 mmol) and 18-crown-6 (190 mg, 0.72 mmol), under N_2_, was added potassium *t*-butoxide (4.03 g, 35.90 mmol) and the reaction heated to 65 °C for 16 h. The reaction mixture was cooled to RT before being diluted with pet ether, washed with water, sat NaHCO_3_, dried (MgSO_4_) and concentrated under vacuum. The crude product was purified on fluorosil (100% pet ether) to give (*E*)-β-farnesene (0.69 g, 95% yield) as a colourless oil.^1^H-NMR (CDCl_3_, 500 MHz): δ 6.41 (dd, 1H, H_2_C=C**H**C, *J* = 17.7 and 10.8 Hz), 5.28 (d, 1H, **H**_2_C=CHC, *J* = 17.7 Hz), 5.19 (m, 1H, RC(Me) = C**H**), 5.13 (m, 1H, (Me)_2_C=C**H**), 5.09 (d, 1H, **H**_2_C=CHC, *J* = 10.9 Hz), 5.04 (m, 2H, H_2_C=CHC(C**H**_2_), 2.28–2.19 (m, 4H), 2.10 (m, 2H), 2.02 (m, 2H), 1.71 (s, 3H), 1.63 (m, 6H); ^13^C-NMR (CDCl_3_, 125 MHz): δ 146.21, 139.03, 135.37, 131.23, 124.39, 124.05, 115.55, 112.96, 39.69, 31.49, 26.74, 26.67, 25.61, 17.62, 15.98).

### Synthetic Blend Formulation

Synthetic blends of EAG-active compounds required for oviposition assays were formulated into polyethylene vial dispensers (6 mm i.d. × 37 mm height, 1 mm wall thickness; Just Plastics, UK) with a piece of cotton bud (ca. 0.125 cm^3^). The ratios of neat compounds administered onto the cotton buds were based on their ratios in volatile extracts as determined by GC-FID. Amounts of compounds used for the floral blend formulation in each dispenser were: benzaldehyde 10.0 mg, benzyl alcohol 0.4 mg, acetophenone 5.0 mg, 3-vinylbenzaldehyde 0.8 mg, (*E*)-cinnamaldehyde 4.0 mg. Amounts of compounds used for the HIPV blend formulation in each dispenser were: (*E*)-DMNT 9.5 mg, n-hexyl acetate 0.4 mg, indole 0.3 mg, (*RS*)-linalool 0.4 mg, (*RS*)-1-octen-3-ol 0.3 mg. Formulated dispensers were entrained over periods of 3 h and 48 h in glass chambers (100 mm i.d. × 60 mm height) at 22 °C/50% RH and extracts analyzed by GC-FID to determine emission rates. The release rate of the synthetic blends: benzaldehyde 11.13 ng/h, benzyl alcohol 3.4 ng/h, acetophenone 5.23 ng/h, 3-vinylbenzaldehyde 6.76 ng/h, (*E*)-cinnamaldehyde 4.46 ng/h; and (*E*)-DMNT 2308.37 ng/h, n-hexyl acetate 142.86 ng/h, indole 194.33 ng/h, (*RS*)-linalool 184.03 ng/h, (*RS*)-1-octen-3-ol 190.03 ng/h.

### Oviposition Assays

To assess oviposition preferences of female *M. vitrata*, dual-choice experiments were carried out in netted cages (120 × 90 × 90 cm) in a greenhouse at KNUST (12:12 h L:D, temperature 34:23 °C L:D, humidity 47:78% L:D). In each replicate of each experiment, ten 5-day-old mated female moths were released into the cages. For the first set of oviposition experiments, moths were offered a choice between a flowering and a non-flowering (3-weeks old) cowpea plant (*n* = 5). In the second set of oviposition experiments, moths were offered a choice between an intact flowering plant and one fed upon by ten 3rd instar *M. vitrata* larvae (larvae remained on the infested plant during the experiment) (*n* = 13). In the third set of oviposition experiments, the choice between a non-flowering cowpea plant plus the synthetic floral blend and an intact flowering plant was offered. The synthetic floral blend was loaded into the dispenser vial, and the lure was mounted on a small wooden stick in the same plant pot (*n* = 6). For the fourth set of oviposition assays, the choice between a flowering cowpea plant plus the HIPV blend and an intact flowering plant was offered. The synthetic HIPV blend was loaded into the dispenser vial, which was mounted on a small wooden stick in the same plant pot (*n* = 11). The number of eggs laid on each plant after 48 h was counted and analyzed by Mann-Whitney U test (two-tailed, α = 0.05) (GenStat version 16).

### Parasitoid Behaviour Assays

Responses of *A. taragamae* and *P. syleptae* to selected identified compounds produced both by damaged cowpea and *S. cannabina* plants were investigated using a glass Y-tube olfactometer (16 mm i.d × 50 mm × 60 mm) (B. J. Pye, Kings Walden, UK). Air, purified by passing through an activated carbon filter at a flow rate of 3 L/min, was divided in two and each air stream passed through the odour sources administered onto filter paper inside a connector, and then into the olfactometer arms. A fluorescent lamp was placed above the olfactometer at a height of 40 cm to provide uniform lighting. Three-day-old naive mated females of *A. taragamae* and 2-day-old naive mated females of *P. syleptae* were introduced individually at the side entry of the olfactometer, and their responses to odour treatments monitored for 10 min. Female wasps not moving away for about 5 min from the release point were discarded. Twenty replications of each test compound at each dose were done with *A. taragamae*, and 50 replications with *P. syleptae*. The position of the odour treatment was altered after every five replications to eliminate any bias in the experimental setup. Parasitoids that did not move up to 1/3 of the chosen arm were considered as non-responders and were not included in the statistical analysis. Differences between the number of parasitoids choosing the control (hexane) and the treated arm were tested by chi-square test, with the null hypothesis that the distribution of wasps over the two arms of the y-tube olfactometer was 50:50 (R Development Core Team 2018).

## Results

### M. Vitrata Oviposition Attractants

**Fig. 1:**
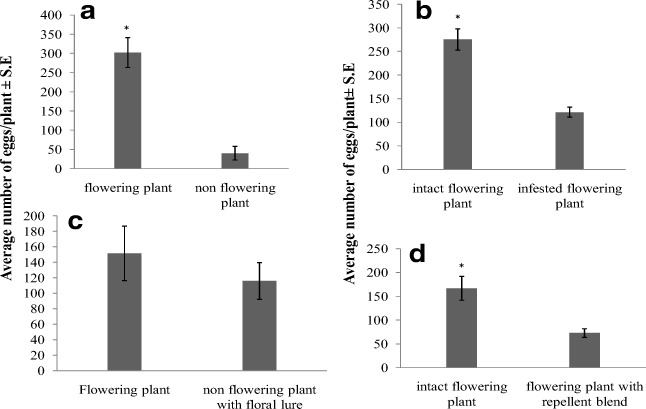
Mean (±SEM) number of eggs laid **a**) on flowering and non-flowering cowpea plants in cage choice bioassays (*n* = 5); **b**) on intact flowering cowpea plants and on those infested by *M. vitrata* larvae (*n* = 13); **c**) on flowering cowpea plants and on non-flowering plants treated with a blend of synthetic floral volatiles (*n* = 6); d) on flowering cowpea plants and on flowering plants treated with a blend of synthetic HIPVs (*n* = 11)

Female moths laid more eggs on flowering cowpea plants than on non-flowering ones (medians: flowering plants: 320, non-flowering plants: 31; Mann–Whitney *U* = 0.0, n_1_ = n_2_ = 5, *p* = 0.008) (Fig. 1a). As it was hypothesized that floral volatiles attract ovipositing moths to larval food plants, cowpea flower headspace extracts were analyzed by coupled GC-electrophysiology (GC-EAG), using female moth antennae. Five peaks elicited consistent antennal responses, which were identified by coupled GC-MS and GC peak enhancement as benzaldehyde, benzylalcohol, acetophenone, a vinylbenzaldehyde isomer and (*E*)-cinnamaldehyde (Supplementary Fig. 1). A synthetic blend of these compounds (using commercially available 3-vinylbenzaldehyde) rendered non-flowering cowpea plants as attractive for ovipositing female moths as flowering plants (medians: flowering plants 150.5, treated plants 92; Mann–Whitney *U* = 15.0, n_1_ = n_2_ = 6, *p* = 0.699) (Fig.1c).

### M. Vitrata Oviposition Repellents

In behavioural assays, the number of eggs laid on uninfested flowering plants was higher than that on plants infested by conspecific larvae (medians: uninfested plants: 275, infested plants 110; Mann–Whitney *U* = 6.0, n_1_ = n_2_ = 13, *p* < 0.001) (Fig. 1b). GC analysis revealed that infested flowers emitted the same compounds as intact ones, but in lower quantities, except the vinylbenzaldehyde isomer (Table 1). However, GC-EAG analysis of headspace extracts of infested leaves located electrophysiologically active peaks, which were subsequently identified as (*R* or *S*)-1-octen-3-ol, *n*-hexyl acetate, (*R* or *S*)-linalool, (*E*)-DMNT and indole (Supplementary Fig. 1). When intact flowering cowpea plants were treated with a synthetic blend of these HIPVs, females laid fewer eggs on treated plants than on flowering plants (medians: flowering plants 161, treated plants 71; Mann–Whitney *U* = 17.0, n_1_ = n_2_ = 11, *p* = 0.003) (Fig.1d). Mechanical damage to leaves caused the induction of (*E*)-2-hexenal, (*Z*)-3-hexen-1-ol and (*Z*)-3-hexenyl acetate, but not of the compounds induced by caterpillar feeding (Table 2). The volatile profile of *S. cannabina* plants infested by *M. vitrata* larvae showed similarities to that of infested cowpea plants, including the emission of 1-octen-3-ol, (*E*)-ocimene, linalool, (*E*)-DMNT, methyl salicylate, indole and (*E*)-nerolidol (Table 2).

**Table 1:** Mean (±SEM) amounts (ng/day) of volatiles released by intact and *M. vitrata* caterpillar-infested cowpea flowers (*Vigna unguiculata* v. Padi-tuya) (*n* = 4). *P* values from two-sample *t*-test

Compound	Intact flower	Infested flower	*P* value
Benzaldehyde	0.254 ± 0.062	0.011 ± 0.001	<0.001
Benzyl alcohol	0.005 e± 0.003	0.00033 ± 0.0003	0.048
Acetophenone	0.095 ± 0.032	0.005 ± 0.003	0.008
Vinylbenzaldehyde isomer	0.011 ± 0.005	0.011 ± 0.007	0.998
(*E*)-Cinnamaldehyde	0.113 ± 0.028	0.013 ± 0.001	0.001

**Table 2:** Mean (±SEM) amounts (ng/day) of volatiles released by intact, *M. vitrata* caterpillar-infested and mechanically damaged cowpea (*Vigna unguiculata* v. Padi-tuya) plants (n = 4), as well as intact and *M. vitrata* caterpillar-infested *Sesbania cannabina* plants (*n* = 3). (*E*)-DMNT = (*E*)-4,8-Dimethyl-1,3,7-nonatriene. Dashes indicate the absence of a compound. KI=Kováts index

Compound	KI^a^	*V. unguiculata*			*S. cannabina*	
		Intact	Caterpillar-infested	Mechanical damage	Intact	Caterpillar-infested
(*E*)-2-Hexenal	823	–	0.011 ± 0.002	0.026 ± 0.014	–	–
(*Z*)-3-Hexen-1-ol	841	–	0.041 ± 0.020	0.131 ± 0.033	–	–
(*R* or *S*)-1-Octen-3-ol	964	–	0.024 ± 0.001	–	–	0.109 ± 0.033
Myrcene	983	–	0.054 ± 0.023	–	–	–
(*Z*)-3-Hexenyl acetate	987	–	0.199 ± 0.018	0.352 ± 0.131	–	–
*n*-Hexyl acetate	996	–	0.017 ± 0.007	–	–	–
(*R* or *S*)-Limonene	1024	–	0.030 ± 0.010	–	–	–
(*E*)-Ocimene	1041	–	0.014 ± 0.001	–	–	0.098 ± 0.04
Nonanal	1084	–	–	–	0.098 ± 0.011	0.088 ± 0.021
(*R* or *S*)-Linalool	1083	–	0.023 ± 0.004	–	–	1.904 ± 0.674
(*E*)-DMNT	1106	–	0.715 ± 0.359	–	–	2.714 ± 0.994
Methyl salicylate	1171	–	0.010 ± 0.001	–	–	0.157 ± 0.08
Decanal	1185	–	–	–	0.097 ± 0.004	0.047 ± 0.023
Indole	1252	–	0.050 ± 0.015	–	–	2.024 ± 0.955
(*E*)-β-Farnesene	1450	–	0.170 ± 0.037	–	–	–
(*E*)-Nerolidol	1551	–	0.022 ± 0.005	–	–	0.232 ± 0.078
*β*-Sesquiphellandrene^b^	1442	–	0.147 ± 0.046	–	–	–
(*Z*)-β-Farnesene^b^	1452	–	–	–	–	0.096 ± 0.016
Linalool oxide^b,c^	1061	–	–	–	–	0.057 ± 0.036

^a^on a non-polar GC column (HP-1)

^b^Tentative identification by GC-MS

^c^furanoid

### Parasitoid Responses to Synthetic Compounds

**Fig. 2:**
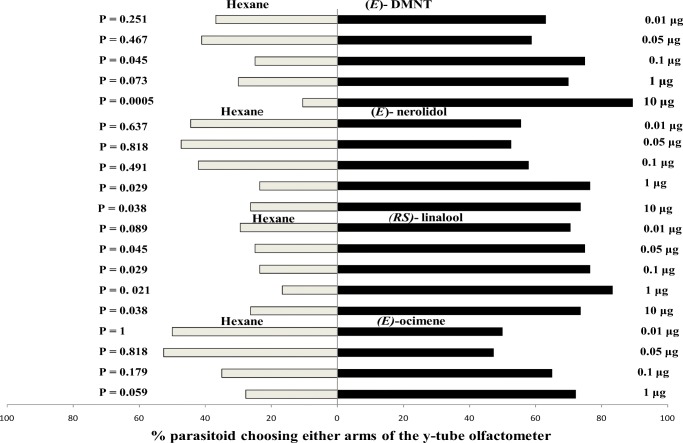
Behavioural responses of 3-day-old naive mated female *A. taragamae* to different doses of synthetic (*E*)-ocimene, (*E*)-nerolidol, (*RS*)-linalool and (*E*)-DMNT in Y-tube olfactometer bioassays. *N* = 20

**Fig. 3:**
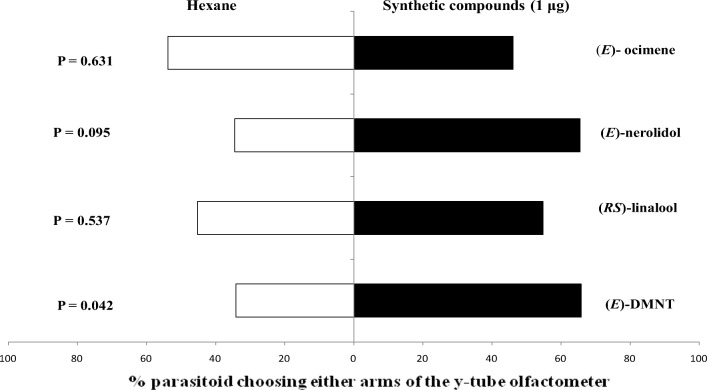
Behavioural responses of naive mated female *P. syleptae* to synthetic (*E*)-ocimene, (*E*)-nerolidol, (*RS*)-linalool and (*E*)-DMNT in Y-tube olfactometer bioassays. *N* = 50

Three-day-old mated female *A. taragamae* wasps did not choose the olfactometer arm treated with any doses of (*E*)-ocimene over the hexane control (Fig. 2). However, they responded positively to (*RS*)-linalool applied at doses of 0.05, 0.1, 1 and 10 μg, but not at 0.01 μg. Positive responses to (*E*)-nerolidol at doses of 1 and 10 μg, but not at 0.1, 0.05 and 0.01 μg, were recorded. (*E*)-DMNT at doses of 0.1 and 10 μg was preferred by wasps to the hexane control, however, they did not discriminate between (*E*)-DMNT and the control at 1, 0.05 and 0.01 μg doses. Female *P. syleptae* wasps did not respond positively to (*E*)-ocimene, (*E*)-nerolidol and (*RS*)-linalool when exposed to 1 μg of each compound in the Y-tube olfactometer. However, there was a significant preference for (*E*)-DMNT (Fig. 3).

## Discussion

In this study, we confirmed female *M. vitrata.* oviposition preference for host, ie. cowpea, plants, at the generative (flowering) stage. Wang et al. (2014) showed that a synthetic blend of nine EAG-active volatile compounds identified from cowpea flowers elicited attraction from *M. vitrata* females in wind tunnel bioassays. Apart from benzaldehyde and acetophenone, composition of their synthetic floral blend differed from that described here, probably because Wang et al. (2014) used a different cowpea cultivar. Coupled GC-EAG analysis in our study located physiologically active components in flower headspace extracts of the Padi-tuya cultivar, and bioassays showed that a synthetic blend of compounds comprising benzaldehyde, benzylalcohol, acetophenone, 3-vinylbenzaldehyde and (*E*)-cinnamaldehyde made flowerless plants just as attractive to ovipositing *M. vitrata* as flowering plants. This suggests that the mixture of these floral volatiles can be used as an olfactory cue by *M. vitrata* to locate host plants for oviposition and larval development. The blend constituents are widespread floral compounds in angiosperms (Knudsen et al. 2006) and attractive to many insects (e.g. Lohonyai et al. 2019; Beck et al. 2012; Tóth et al. 2011), whereas a vinylbenzaldehyde isomer different from the one in this study has been isolated from *N. tabacum* and found to be electrophysiologically active on a particular type of olfactory receptor neuron of *Helicoverpa armigera* Hübner and *H. virescens* (Lepidoptera: Noctuidae) (Røstelien et al. 2005).

Florivory can negatively affect plant reproductive success (McCall and Irwin 2006), and altered volatile profiles may change the dynamics of plant-pollinator interactions (Lucas-Barbosa et al. 2011). However, only a few studies have determined the emission of floral volatiles after flower damage by herbivores. For example, damage to flowers of *Pastinaca sativa* L. (Apiaceae) by *Depressaria pastinacella* Duponchel (Lepidoptera: Elachistidae) causes increase in the quantity of floral compounds emitted, particularly octyl esters (Zangerl and Berenbaum 2009). In contrast, we observed a significant decrease in overall floral volatile emission after florivory by *M. vitrata* larvae. As *M. vitrata* is not a cowpea pollinator, such a response may make cowpea plants less apparent to ovipositing moths, thus reducing further damage to generative organs and preserving fitness of the individual. Kessler et al. (2019) showed that floral volatile emissions can be context-dependent: benzylacetone attracts key pollinators (hawkmoths) of *N. attenuata* Torr ex *S. Watson,* but repels florivores (a leaf beetle species).

*M. vitrata* larvae not only feed on generative organs, but also on tender leaves and terminal shoots of cowpea (Bendera et al. 2015; Sharma et al. 1999). When five-day-old mated *M. vitrata* females were presented with a choice between non-infested flowering cowpea plants and those infested by conspecific larvae, moths preferred to lay eggs on non-infested ones. GC analysis of leaf headspace extracts of infested plants revealed enhanced levels of fifteen compounds. Of these, a synthetic blend of the electrophysiologically active (*E*)-DMNT, indole, n-hexyl acetate, (*RS*)-1-octen-3-ol and (*RS*)-linalool reduced oviposition rates on flowering cowpea, indicating that the blend was repellent. Tissue damage by pest insects often stimulates the release of herbivore-induced plant volatiles (HIPVs) that can deter further herbivore attack (Turlings and Erb 2018). De Moraes et al. (2001) observed that *H. virescens* moths were repelled by HIPVs from their larval host and argued that this recognition of HIPVs may reduce negative fitness consequences posed by competitors. In another study, when transgenic *N. tabacum* plants, which produced high amounts of linalool, and non-transformed control plants where presented to *H. armigera* for oviposition, fewer eggs were laid on transgenic plants, suggesting a repellent role for linalool (McCallum et al. 2011). Anastasaki et al. (2018) showed that *Tuta absoluta* Meyrick (Lepidoptera: Gelechiidae) females preferred to oviposit on healthy tomato (*Solanum lycopersicum* L.) (Solanaceae) plants as opposed to those infested by conspecific larvae, and assigned EAG activity to some of the induced volatiles. In our studies, (*E*)-DMNT was the most abundant compound collected from damaged cowpea leaves, which was also reported to be the major cowpea leaf volatile constituent upon infestation by *Spodoptera frugiperda* J. E. Smith (Lepidoptera: Noctuidae) (Carroll et al. 2008). Interestingly, in this case, *S. frugiperda* moths were attracted by conspecific-induced plant volatiles, especially DMNT, which highlights the fact that HIPVs play different roles in different plant-insect interactions, i.e. the effect of plant volatiles is context-dependent (He et al. 2019).

The observed difference in the leaf volatile profile between plants damaged by *M. vitrata* caterpillars and mechanically damaged ones highlights the role of herbivore-associated elicitors in inducing volatile emission patterns (Bonaventure et al. 2011). When cowpea leaves were fed upon by *M. vitrata* larvae, the emission of fifteen volatiles was induced, whereas mechanical damage only caused the enhanced emission of (*E*)-2-hexenal, (*Z*)-3-hexen-1-ol and (*Z*)-3-hexenyl acetate. Thus, it is highly likely that elicitors in the oral secretion of *M. vitrata* caterpillars are introduced into the feeding wounds, which then results in the upregulation of production of a range of compounds. The identity of such elicitors remains to be revealed.

HIPVs can not only repel the herbivores themselves, but can also attract their natural enemies, such as parasitic wasps (Turlings and Erb 2018). For example, the push/pull system in maize in East Africa utilizes a cropping regime, where trap plants on the perimeter lure maize pests (Lepidoptera: Noctuidae) away from the main crop by means of attractive semiochemicals, whereas intercropped plants repel the moths and attract their parasitoids via a suite of volatile terpenes (Khan et al. 2008). De Moraes et al. (2001) argue that HIPV blends are not only utilized by certain herbivorous insects to avoid competitors at oviposition sites, but also to avoid potential attack by natural enemies attracted by the same volatile blend. Of the parasitoids of *M. vitrata*, Dannon et al. (2010) showed that *A. taragamae,* a solitary endoparasitoid, preferred volatiles from *M. vitrata*-infested cowpea plants, as compared to blank air, in olfactometer bioassays, although the compounds mediating this interaction were not determined. The preference of this parasitic wasp for the odour of infested plants has also been investigated in the context of another host species (Nurkomar et al. 2017). Similarly, Souna et al. (2019) demonstrated the attraction of *Therophilus javanus* Bhat & Gupta (Hymenoptera: Braconidae) to the odour of *M. vitrata* caterpillar-damaged cowpea flowers and pods.

As *A. taragamae* is reported to parasitize 63% of *M. vitrata* larvae feeding on *S. cannabina* (Huang et al. 2003), we examined the behavioural response of this wasp to four synthetic semiochemicals produced by both cowpea and *S. cannabina* upon *M. vitrata* infestation. In general, wasps responded most sensitively to (*RS*)-linalool and (*E*)-DMNT in a positive manner, followed by (*E*)-nerolidol, whereas (*E*)-ocimene was not active in these tests. *P. syleptae,* an egg parasitoid of *M. vitrata,* only showed preference for (*E*)-DMNT, although the four synthetic compounds were tested only at a single dose. These indicate that the two parasitoids potentially exploit host-related HIPVs to locate *M. vitrata* caterpillars and eggs. In the case of the egg parasitoid, *P. syleptae* may search for caterpillar feeding sites by means of HIPVs, where it has a greater chance of finding host eggs on non-infested neighbouring plants. Alternatively, *M. vitrata* egg deposition may induce the release of HIPVs, including (*E*)-DMNT, as part of the cowpea plant’s response to herbivore attack, a phenomenon described from maize landraces (Tamiru et al. 2011). The above four compounds not only elicit antennal responses from parasitic wasps in other plant-herbivore systems (Gouinguene et al. 2005), but also attract them (e.g. Kos et al. 2013; Krugner et al. 2014; Li et al. 2018).

## Conclusion

In summary, the work in this study has demonstrated that floral volatiles guide host-searching *M. vitrata* females to cowpea plants for oviposition, whereas the volatile blend emitted after caterpillar feeding repels ovipositing moths. Also, certain constituents of the repellent blend are behaviourally preferred by natural enemies of *M. vitrata*, indicating they may be attractive. Therefore, the synthetic blends have the potential to be incorporated in future IPM strategies to reduce *M. vitrata* damage in cowpea by both repelling moths out of the main crop and attracting them to trap crops/into traps, as well as manipulating the density of natural enemies around/within the crop for biological control. To demonstrate the suitability of these semiochemicals for *M. vitrata* management, further assays comparing the activity of synthetic blends with live plants, followed by field trials testing different formulations, will be necessary.

## Electronic supplementary material

Supplementary Fig. 1 (DOCX 62 kb)
