# Supplementary material for: Identification of Semiochemicals from Cowpea, Vigna unguiculata, for Low-input Management of the Legume Pod Borer, Maruca vitrata
Source: J Chem Ecol. 2020 Jan 17;46(3):288–98. doi: 10.1007/s10886-020-01149-7 (PMC7142049; doi:10.1007/s10886-020-01149-7)
Supplement: Supplementary file 1 — (DOCX 62 kb) [file 10886_2020_1149_MOESM1_ESM.docx]

**
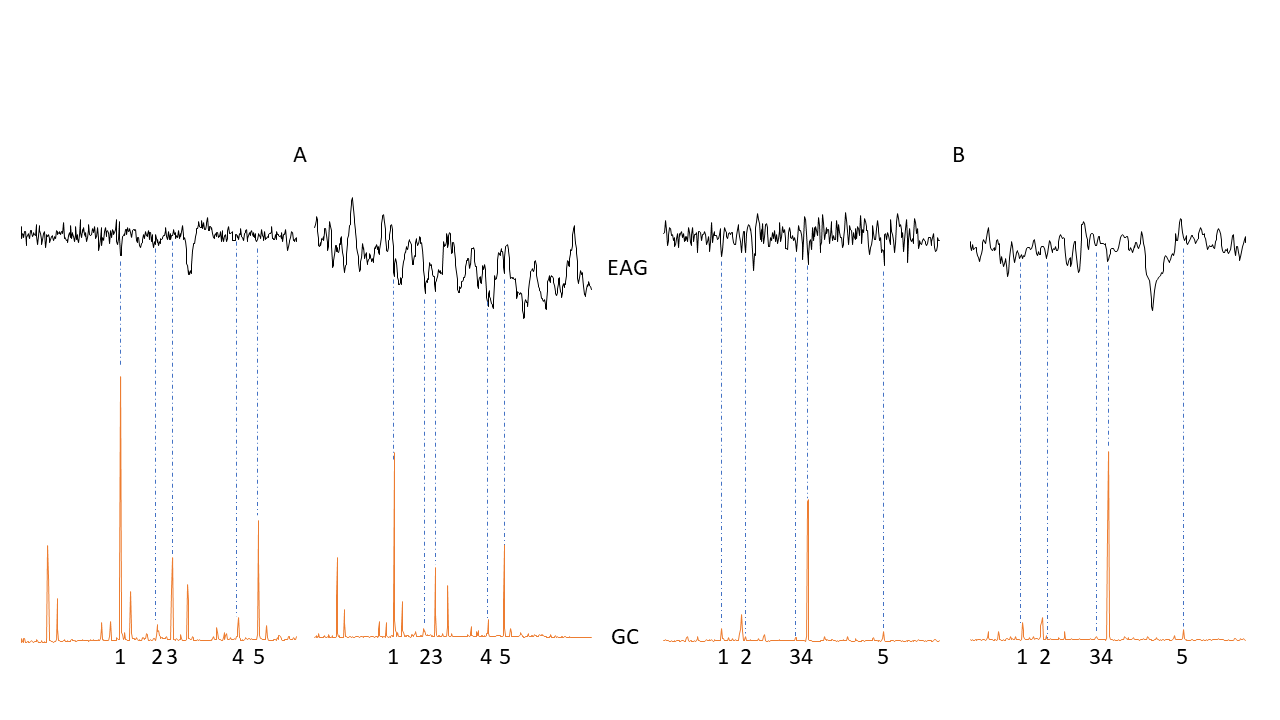
**

**Supplementary Fig. 1.** Typical coupled gas chromatography-electrophysiology (GC-EAG) recordings from the antennae of female *Maruca vitrata* to cowpea volatile extracts. Numbers indicate EAG-active peaks. A: floral volatile extract, 1: benzaldehyde, 2: benzylalcohol, 3: acetophenone, 4: vinylbenzaldehyde isomer, 5: (*E*)-cinnamaldehyde. B: HIPV extract, 1: (*R* or *S*)-1-octen-3-ol, 2: n-hexyl acetate, 3: (*R* or *S*)-linalool, 4: (*E*)-DMNT, 5: indole.
